# Supplementary material for: An evaluation tool to strengthen the collaborative process of the public-private partnership in the veterinary domain
Source: PLoS One. 2021 May 28;16(5):e0252103. doi: 10.1371/journal.pone.0252103 (PMC8162688; doi:10.1371/journal.pone.0252103)
Supplement: S1 File — (PDF) [file pone.0252103.s001.pdf]

PPP EVALUATION TOOL

Introduction and presentation of the developed tool

Thank you for your willingness to participate in our study!
As already mentionned, this questionnaire will require you about 1 hour to complete. You can stop at anytime and come back to the questionnaire later on using the same device. Most of the questions are compulsory, you will need to answer them before moving to another page of the questionnaire.

Public-Private Partnership (PPP) in the veterinary domain is defined by the OIE as “a joint approach in which the public and private sectors agree responsibilities and share resources and risks to achieve common objectives that deliver benefits in a sustainable manner”.

In connection with the OIE Public-Private Partnership initiative, a tool for a self-evaluation of the quality of the PPP process is being developed by CIRAD (French Research Institute for Agricultural Development), as part of my PhD thesis.

This evaluation tool may also be used in the context of the PPP targeted support of the OIE PVS (Performance of Veterinary Services) Pathway, currently under development.

The objectives of the tool are:
-To characterize the PPP organization process
-To evaluate PPP’s strengths and weaknesses and how the process influences the quality of the PPP
-To provide meaningful recommendations to improve the PPP process at all stages of implementation: during the design, implementation and after the end of the PPP to learn lessons

The evaluation criteria included in the tool were selected based on literature, on the work conducted by OIE on PPP best practices and benefits/impacts in the veterinary domain and on three in-depth case-studies (Ethiopia, Indonesia, Paraguay). The list of criteria was adjusted and validated during four OIE PPP regional workshops, conducted in 2019 in Africa and Asia, bringing together stakeholders from both public and private sectors. The evaluation tool was also field-tested on PPP in Paraguay.

The developed tool consists of 48 evaluation criteria divided into 9 sections. Each section is assessed using 3 to 9 criteria. The criteria influence 6 quality attributes, as shown in the following figure:

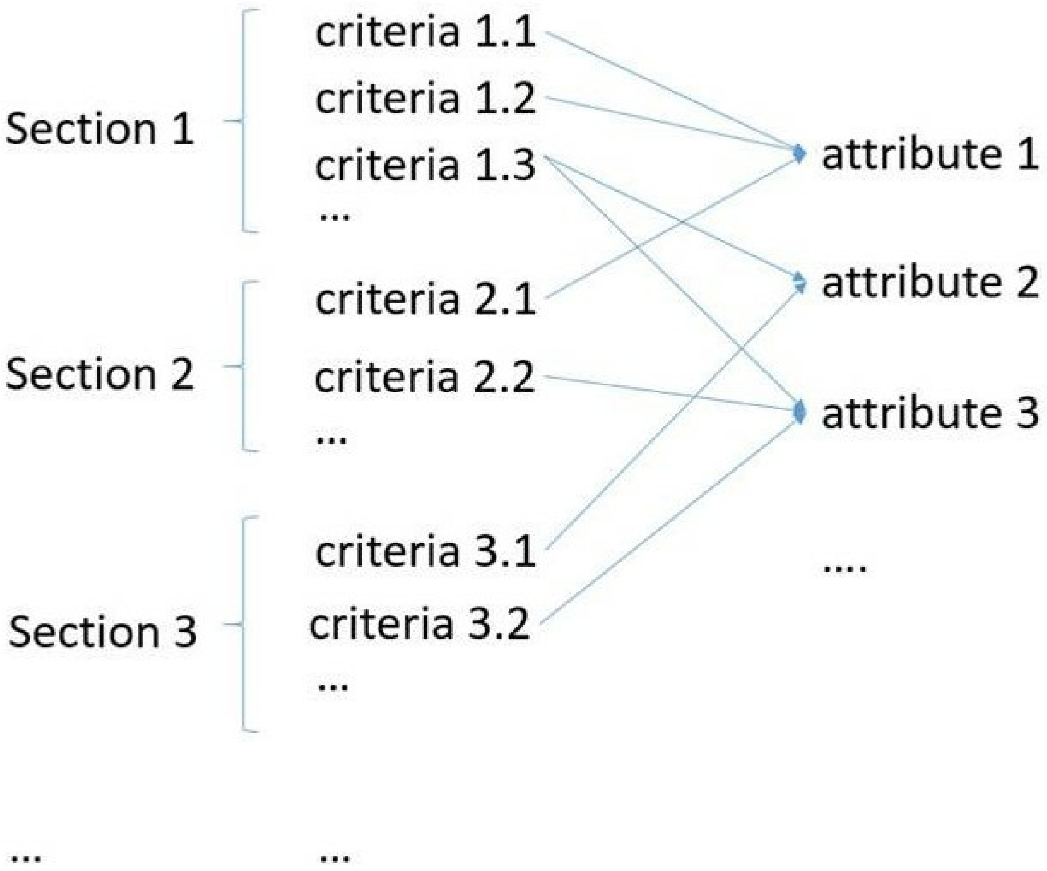

Each of the 9 sections represents a critical component of a PPP process, as defined in the OIE PPP Best practices handbook, including:
1-Common objectives;
2-Specific interests/benefits and risks/constraints;
3-Governance and legal framework;
4-Planning and responsibilities;
5-External factors and enabling environment;
6-Competencies and trainings;
7-Communication and transparency;
8-Collaboration and engagement;
9-Evaluation.

Those criteria influence six quality attributes: relevance, adaptability, operationality, stability, inclusiveness and acceptability.

This tool allows for a semi-quantitative evaluation. Indeed, each evaluation criterion is scored from 0 to 3. The scoring of the criteria provides 2 different outputs, as shown in the following figure:

The output 1 represents pie charts for each section to visualize the strengths and weaknesses of the PPP .

The output 2 represents one radar chart showing the influence of the PPP process organisation on quality attributes.

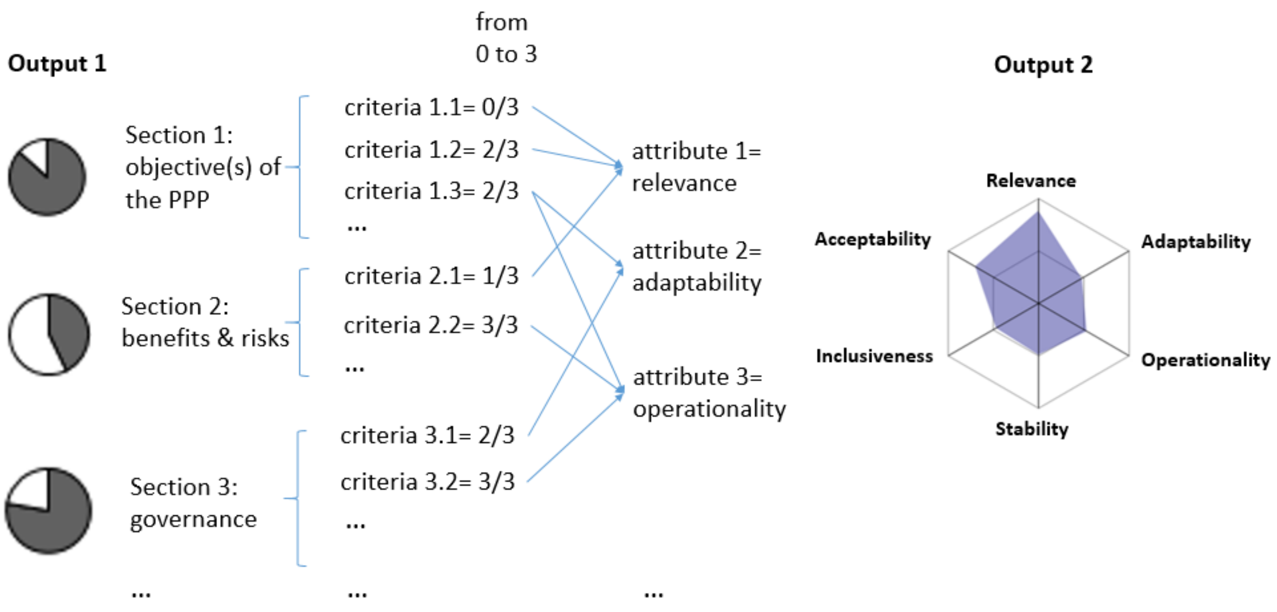

PPP EVALUATION TOOL

Presentation of the questionnaire

The purpose of this questionnaire is to elicit your expertise to refine our selection of criteria and their influence on the quality attributes.

This questionnaire consists of 36 questions, divided in 4 main parts.

Part 1 : Experts

- Background information of the expert (5 questions)

Part 2 : Evaluation criteria

- Section 1: Common objective(s) (2 questions)
- Section 2: Specific interests/benefits and risks/constraints (2 questions)
- Section 3: Governance and legal framework (2 questions)
- Section 4: Planning and responsibilities (2 questions)
- Section 5: External factors and consequences (externalities) (2 questions)
- Section 6: Competencies and trainings (2 questions)
- Section 7: Communication and transparency (2 questions)
- Section 8: Collaboration (2 questions)
- Section 9: Evaluation (2 questions)

Part 3 : Quality attributes

- Validation of the 6 quality attributes (1 questions)

Part 4 : Influence of the criteria on the quality attributes

- Stability attribute (2 questions)
- Relevance attribute (2 questions)
- Operationality attribute (2 questions)
- Acceptability attribute (2 questions)
- Adaptability attribute (2 questions)
- Inclusiveness attribute (2 questions)

The list and the definition of all the criteria are available here.
The list and the definition of all the quality attributes are available here.
You don't need them right away, the definitions will be given to you as the questionnaire

progresses, but downloading these lists can make it easier for you to complete the questionnaire.

PPP EVALUATION TOOL

PART 1 : INFORMATION ON THE EXPERTS

* 1. Please enter your name and surname

* 2. Please check the box(es) that best describes the institution you are currently working for:

- [ ] Public: Veterinary Services
- [ ] Public: Other governmental authority (such as Minsitry of Agriculture)
- [ ] Public: Academia
- [ ] Private: Private veterinarian
- [ ] Private: Veterinary Statutory Body or Veterinary Association
- [ ] Private: Producers organizations
- [ ] Private: Private companies
- [ ] Private: Para-public agency
- [ ] Private: NGO/ Private foundation
- [ ] Intergouvernmental organization (OIE, FAO, IFAD etc.)
- [ ] Other (please specify)

* 3. Please check the box(es) that best describes your field of expertise:

- [ ] Veterinary
- [ ] Biology
- [ ] Epidemiology
- [ ] Economy
- [ ] Sociology
- [ ] Agronomy
- [ ] Ecology
- [ ] Other (please specify)

* 4. What is your role in PPP(s) ?

- ○ Public partner
- ○ Private partner
- ○ Catalyzer
- ○ Other (please specify)

* 5. How long have you been involved in PPP(s)?

- ○ Less than 2 years
- ○ From 2 to 5 years
- ○ From 5 to 10 years
- ○ More than 10 years

PPP EVALUATION TOOL

PART 2 : EVALUATION CRITERIA

In this part, we will ask you to validate or invalidate the evaluation criteria for each of the 9 sections.

Section 1: OBJECTIVE(S) of the PPP.

Please, validate or invalidate the evaluation criteria of the section 1.

* 6. Are all those criteria relevant when it comes to the assessment of the objective(s) of PPP in the veterinary domain?

	Relevant	Not relevant
**1.1 Common objective(s):** All parties (public and private actors) must agree on the definition of the overall objective(s) to be achieved and on the service to be delivered.	◯	◯
**1.2 Formalization of the common objective:** Level of formalization of the common objective of the PPP (MoU, Letter of Agreement, Oral consent, etc.).	◯	◯
**1.3 Relevance of common objective:** Relevance of the common objective(s) regarding the epidemiological, socio-economic, environmental and political (institutional) context.	◯	◯
**1.4 Position of the partners regarding this common objective:** The common objective should be transparent and understood by each partner. It should satisfy each partner regarding his own strategies, needs and benefits.	◯	◯
**1.5 Added-value of the PPP :** The PPP should be an added value to reach the common objective(s) of the program (consideration has been given to conducting this program with a single sector but the objective can not be reach or with more difficulties).	◯	◯

If you selected some criteria as non-relevant, please specify

* 7. Do you identify some missing criteria to assess the objective(s) of PPP in the veterinary domain, or do you have any other comments?

- ○ No
- ○ Yes (please specify)

Section 2 : SPECIFIC INTEREST / BENEFITS AND RISKS/CONSTRAINTS.
Please, validate or invalidate the evaluation criteria of the section 2

* 8. Are all those criteria relevant when it comes to the assessment of the specific interest / benefits and risks/constraints of a PPP in the veterinary domain?

	Relevant	Non relevant
**2.1 The specific interest of the different partners:** The different partners have specific interest and expected benefits to enrol in the partnership. Those specific interests should be explicit, transparent, formalized and should be understood by the other partners.	○	○
**2.2 Repartition of benefits:** The PPP may have differing benefits for the public and private sectors. The partners should feel that the repartition of benefits between the different partners is fair.	○	○
**2.3 Repartition of the other outputs (ownership):** The repartition of the PPP outputs (such as products, intellectual right, property right) should be fair and formalized.	○	○
**2.4 Achievement of goal(s) of the Veterinary Services:** The PPP should help to reach the goal(s) previously defined by the Veterinary Services.	○	○
**2.5 Achievement of goal(s) of the private sector:** The PPP should help to reach the goal(s) previously defined by the private sector.	○	○

	Relevant	Non relevant
**2.6 Risks and constraints:** The different partners could have specific constraints/risks (financial, societal, etc.) by engaging in this PPP: they should be identified, discussed and understood by the partners.	○	○
**2.7 Repartition of the constraints:** The PPP may have differing constraints (financial, societal, etc.) to the public and/or private sectors, and the partners should feel that the repartition of those constraints between the different partners is fair.	○	○
**2.8 Change of practices:** The achievement of the common objective may require a change in the practices (e. g. change in the use of medicines, change in farming techniques, change in vaccination planning, etc.). Those changes should be anticipated, accepted and accompanied if needed.	○	○

If you selected some criteria as non-relevant, please specify

* 9. Do you identify some missing criteria to assess the specific interest / benefits and risks/constraints of a PPP in the veterinary domain, or do you have any other comments?

○ No

○ Yes (please specify)

Section 3: GOVERNANCE AND LEGAL FRAMEWORK of the PPP.
Please, validate or invalidate the evaluation criteria of the section 3

* 10. Are all those criteria relevant when it comes to the assessment of the governance and legal framework of PPP in the veterinary domain?

	Relevant	Not relevant
**3.1 Formalization of the PPP :** The terms of the rationale of the PPP must be set out clearly, either in a formal contract or in an alternative form appropriate to the PPP (MoU, Letter of Agreement, Oral consent etc.).		
**3.2 Knowledge of the terms of the PPP (contract) by the partners :** The different partners should be aware of the terms of the contract and understand them all.		
**3.3 Endorsement by all partners:** The documents where the terms of the PPP are formalized, are endorsed by all partners from different sectors.		
**3.4 Shared decision making process:** Shared decision making with an equality in power relationship can represent a key success factor of the PPP.		
**3.5 International, national or regional laws:** Legal obligations, laws and constraints from the regions or from the country are understood and properly applied by all partner and a public partner is responsible to ensure the application of the laws. The public partner should ensure that the PPP is lawful and that any legal obligations or constraints are understood and properly implemented by all parties.		
**3.6 Adequacy of the objective with the Veterinary Services (VS) mandate:** The public partner(s) must ensure that the service(s) to be delivered falls within their VS statutory or political mandate and meet the intention of that mandate. The mandate should be strengthened. The public sector must assume the entire responsibility of the VS mandate.		

	Relevant	Not relevant
**3.7 Opportunities of private parties' involvement:** The public party should ensure that relevant private partners have equal opportunities for engagement in a new PPP, respecting the country market rules. The public sector should propose a transparent call for the tender process.	◯	◯
**3.8 Funding & human resource availability:** The funding and HR should be available and sufficient. If an external source is providing money, the PPP plans how to be sustainable when the other source will stop.	◯	◯
**3.9 Funding & HR repartition:** The repartition of the funding and HR should be fair.	◯	◯

If you selected some criteria as non-relevant, please specify

* 11. Do you identify some missing criteria to assess the governance and legal framework of a PPP in the veterinary domain, or do you have any other comments?

◯ No

◯ Yes (please specify)

Section 4: PLANNING AND RESPONSIBILITIES of the PPP.
Please, validate or invalidate the evaluation criteria of the section 4

* 12. Are all those criteria relevant when it comes to the assessment of the planning and responsibilities of PPP in the veterinary domain?

	Relevant	Not relevant
**4.1 Division of roles and responsibilities**: The role of each partner should be properly defined. Formalisation of the partner's areas of action in the PPP should be specified in the contract, i.e. the tasks they are assigned regarding collaboration and coordination of PPP.	○	○
**4.2 Existence of organizational chart:** An organizational chart of the PPP itself is a good element to know who depends on whom, who decides for whom.	○	○
**4.3 Potential other partners:** It could be useful/helpful to involve some other partners in the PPP for the stability of the initiative. They could have been identified by the partners.	○	○
**4.4 Inclusion of vulnerable group :** PPPs should enhance equity in terms of his outcomes (economy, health, well-being etc.). This can be done by truly involve the vulnerable group (indigenous, women, young people, etc.) in the PPP or by inviting them during meetings or workshops.	○	○
**4.5 Defined duration:** The duration of the PPP should be predefined by both partners, with the possibility to extend the period or to renew the partnership in predefined renewal conditions (e.g. if deemed appropriate following joint evaluation and review).	○	○
**4.6 Modalities of the PPP:** By proposing a diversity of modalities of application to the partners, the PPP can satisfy a higher number of partners.	○	○

If you selected some criteria as non-relevant, please specify

* 13. Do you identify some missing criteria to assess the planning and responsibilities of PPP in the veterinary domain, or do you have any other comments?

- ◯ No
- ◯ Yes (please specify)

Section 5: EXTERNAL FACTORS AND CONSEQUENCES (EXTERNALITIES) of the PPP.

Please, validate or invalidate the evaluation criteria of the section 5

* 14. Are all those criteria relevant when it comes to the assessment of the the external factors and consequences of PPP in the veterinary domain?

	Relevant	Not relevant
**5.1 Threat of the PPP:** Some external factors related to the context (epidemiological, institutional, socio-economical, environmental risk) can threaten the stability of the PPP.	○	○
**5.2 Infrastructures:** Lack of appropriate infrastructures (such as road, water, electricity, etc.) could represent constraints for the proper implementation of the PPP. However, the PPP can also have the power to improve those failures, to respond to those constraints.	○	○
**5.3 Organisation of private sector :** Lack of organisation of the private sector could represent constraints for the proper implementation of the PPP. However, the PPP can also have the power to improve that organization, to improve the supply chain.	○	○
**5.4 Cost to the society (negative externalities):** Every initiative can have some negative societal externalities (e.g. constraints on a category of partners), economic externalities (e.g. financial competitiveness with other partners, competitiveness for resource) or environmental externalities (contamination)/externalities on biodiversity (loss of wild or domestic animal biodiversity). Those externalities should be anticipated in order to be minimized.	○	○

If you selected some criteria as non-relevant, please specify

* 15. Do you identify some missing criteria to assess the external factors and consequences of a PPP in the veterinary domain, or do you have any other comments?

◯ No

◯ Yes (please specify)

Section 6: COMPETENCIES AND TRAININGS.
Please, validate or invalidate the evaluation criteria of the section 6.

* 16. Are all those criteria relevant when it comes to the assessment of the competencies and trainings process of PPP in the veterinary domain?

	Relevant	Not relevant
**6.1 Confidence in other partner competencies :** The partners should feel confident about their partner competencies to fulfil the common objective(s).	◯	◯
**6.2 Satisfaction of actors about their own competencies:** The different partners should be satisfied of their own competencies to reach the common objective(s); the partners should feel legitimate to enrol their role.	◯	◯
**6.3 Organisation of training:** Trainings well designed and well planned for operating partners shoul be organized if needed.	◯	◯
**6.4 Accessibility and frequencies of trainings :** The training organized should be at an appropriate frequency and should be accessible to all operating partners, to all partners that feel the need to improve their competencies.	◯	◯
**6.5 Reinforcement of the Veterinary Services through trainings :** The Veterinary Services can be reinforced thanks to the PPP through training organized for his staff.	◯	◯

If you selected some criteria as non-relevant, please specify

* 17. Do you identify some missing criteria to assess the competencies and trainings of PPP in the veterinary domain, or do you have any other comments?

◯ No

◯ Yes (please specify)

Section 7: COMMUNICATION AND TRANSPARENCY of the PPP.
Please, validate or invalidate the evaluation criteria of the section 7.

* 18. Are all those criteria relevant when it comes to the assessment of the communication and transparency of PPP in the veterinary domain?

	Relevant	Not relevant
**7.1 Consultation:** The PPP must have an agreed consultation strategy. The frequency of the meetings is to be appreciated according to the need of the partners.	◯	◯
**7.2 Agreement in resolution modalities in case of conflict:** A manner to resolve potential conflict(s) between partners should be identified: which partner/jurisdiction to contact, how to resolve this conflict?	◯	◯
**7.3 Communication with other parties and with end users:** The capability of the PPP to keep other parties informed, in a transparent, effective and timely manner, of PPP activities and programmes.	◯	◯
**7.4 Transparency:** All parties must ensure that the actions of the PPP are developed with appropriate transparency to all stakeholders at every level (repartition of the outputs, of the benefits, repartition of the risk, modalities of action, activities of each partner etc.).	◯	◯

If you selected some criteria as non-relevant, please specify

* 19. Do you identify some missing criteria to assess the communication and transparency of PPP in the veterinary domain, or do you have any other comments?

◯ No

◯ Yes (please specify)

Section 8: COLLABORATION in the PPP.

Please, validate or invalidate the eveluation criteria of the section 8.

* 20. Are all those criteria relevant when it comes to the assessment of the collaboration process in PPP in the veterinary domain?

	Relevant	Not relevant
**8.1 Willingness to collaborate:** The partners should be happy/satisfied to collaborate with their partners and the PPP must have an agreed stakeholder engagement, which includes an appropriate approval process (formalisation of rationale behind the willingness to collaborate for this PPP).	○	○
**8.2 Level of involvement of partners/mobilisation:** Partners should be satisfied about the engagement of other parties in their assigned areas of action, role and responsibilities in the PPP.	○	○
**8.3 Existence of champions:** The existence of champion(s), who has experience in PPPs and promoting the well functionning of PPP at national, regional or local level, can represent a key success factor of the PPP.	○	○
**8.4 Actors' acceptations of their own roles:** The different partners should be satisfied with their own roles in the partnership and their tasks and with the recognition of his role by the other partners.	○	○

If you selected some criteria as non-relevant, please specify

* 21. Do you identify some missing criteria to assess the collaboration of PPP in the veterinary domain, or dou you have any other comments?

◯ No

◯ Yes (please specify)

Section 9: EVALUATION of the PPP.
Please, validate or invalidate the evaluation criteria of the section 9.

* 22. Are all those criteria relevant when it comes to the assessment of the evaluation process of PPP in the veterinary domain?

	Relevant	Not relevant
**9.1 Internal evaluations of the PPP:** The different partners of the PPP should regularly monitor the advancement of the program and discuss about the main conclusion and the way to improve the PPP.	◯	◯
**9.2 Choices of indicators for internal evaluations:** The partners must agree on how the PPP is monitored, and on the choices of indicators for internal evaluation.	◯	◯
**9.3 External evaluations:** External evaluations help to promote positive changes in the PPP. Partners must agree on how the PPP is monitored, and on the choices of indicators for external evaluations.	◯	◯

If you selected some criteria as non-relevant, please specify

* 23. Do you identify some missing criteria to assess the evaluation process of PPP in the veterinary domain, or do you have any other comments?

- ◯ No
- ◯ Yes (please specify)

cirad

PPP EVALUATION TOOL
PART 3 : QUALITY ATTRIBUTES

Validation of the quality attributes.
Please, validate or invalidate the six quality attributes of a PPP.

* 24. Are the six attributes relevant to the evaluation of the quality of the PPP process?

	Relevant	Not relevant
**Stability** The collaboration process in the PPP is stable in the time defined by the stakeholders, and thus despite the potential changing environment. The formalisation and endorsement of the agreement satisfied all relevant stakeholders.	◯	◯
**Relevance** PPP strategy, modalities and activities are relevant regarding the main objective. The main objective is relevant regarding the context (epidemiological, institutional, environmental, societal). PPP represent a clear added value to achieve the objective.	◯	◯
**Operationality** The governance of PPP is operational, and collaboration is effectively implemented to meet the main objective. Roles in PPP are adequately allocated to actors with regard to their mandates and competencies. Trainings are organised to be sure that stakeholders can fit their roles. The mechanisms for the resources allocation are defined. The resources are appropriate and available for the effective implementation of activities.	◯	◯

	Relevant	Not relevant
**Acceptability** All relevant stakeholders demonstrate trust into the PPP, mutual understanding and willingness to collaborate. The objectives and outcomes of the PPP meet the stakeholder's expectations. Actors are satisfied with the repartition of resources. The PPPs have a societal legitimacy.	○	○
**Adaptability** PPP can adapt and evolve upon changes in governance modalities, organizational process and other structural modalities in order to best suit the changing environment.	○	○
**Inclusiveness** Relevant actors participate in governance mechanisms and decision making process. PPP provide a trustworthy environment where stakeholders can freely express their views and be heard, creating mutual understanding. New partners are considered if relevant. The vulnerable group(s) are take into consideration.	○	○

If you have selected some attributes as non-relevant, or if you have any comments, please specify:

PPP EVALUATION TOOL

PART 4 : INFLUENCE OF THE CRITERIA ON THE QUALITY ATTRIBUTES

Please find a reminder of the criteria definition here

Influence of the evaluation criteria on the STABILITY attribute

STABILITY : The collaboration process in the PPP is stable in the time defined by the stakeholders, and thus despite the potential changing environment. The formalisation and endorsement of the agreement satisfied all relevant stakeholders.

Please, indicate how the selected criteria influence the STABILITY attribute and your degree of certainty about this influence.

* 25. Does the following PPP process criteria influence the STABILITY of the PPP?

	Influence level	How confident are you with your answer?
1.2 Formalization of the common objective		
1.5 Added-value of the partnership		
2.6 Risk and constraints		
3.1 Formalization of the PPP		
3.2 Knowledge of the terms of the PPP (contract) by the partners		
3.3 Endorsement by all partners		
3.8 Funding abd human resource availability		
4.3 Potential other partners		
4.5 Defined durations		
4.6 Modalities of the PPP		
5.1 Threat of the PPP		
5.2 Infrastructures		
5.3 Organisation of the private sector		
5.4 Cost to the society (negative externalities)		
7.2 Agreement in resolution modalities in case of conflict		
7.4 Transparency		

* 26. Do you identify some missing criteria presented in the sections 1 to 9 (available here) that can influence the STABILITY attribute, or do you have any other comments?

◯ No

◯ Yes (please specify which one(s))

Influence of the evaluation criteria on the RELEVANCE attribute.
RELEVANCE: PPP strategy, modalities and activities are relevant regarding the main objective. The main objective is relevant regarding the context (epidemiological, institutional, environmental, societal). PPP represent a clear added-value to achieve the objective.

Please, indicate how the selected criteria influence the RELEVANCE attribute and your degree of certainty about this influence.

* 27. Does the following PPP process criteria influence the RELEVANCE of the PPP?

	Level of influence	How confident are you with your answer?
1.3 Relevance of the common objective and of the strategy regarding the context		
1.5 Added-value of the PPP		
2.1 The specific interest of the different partners		
2.4 Achievement of goal(s) of the Veterinary Services		
2.5 Achievement of goal(s) of the private sector		
3.6 Adequacy of the objective with the Veterinary Services mandate		
5.4 Cost to the society (negative externalities)		
6.5 Reinforcement of the Veterinary Services through trainings		

* 28. Do you identify some missing criteria presented in the sections 1 to 9 (available here) that can influence the RELEVANCE attribute, or do you have any other comments?

◯ No

◯ Yes (please specify which one(s))

Influence of the evaluation criteria on the OPERATIONALITY attribute.
Operationality: The governance of PPP is operational, and collaboration is effectively implemented to meet the main objective. Roles in PPP are adequately allocated to actors with regard to their mandates and competencies. Trainings are organised to be sure that stakeholders can fit their roles. The mechanisms for the resources allocation are defined. The resources are appropriate and available for the effective implementation of activities.

Please, indicate how the selected criteria influence the OPERATIONALITY attribute and your degree of ccertainty about this influence.

* 29. Does the following PPP process criteria influence the OPERATIONALITY of the PPP?

	Level of influence	How confident are you with your answer?
1.1 Common objective(s)		
2.8 Change of practices		
3.5 International, national or regional laws		
3.8 Funding & human resource availability		
4.1 Division of roles and responsibilities		
4.5 Predefined duration		
5.1 Threat of the PPP		
5.2 Infrastructures		
5.3 Organisation of private sector		
6.3 Organisation of training		

	Level of influence	How confident are you with your answer?
6.4 Accessibility and frequencies of trainings		
7.1 Consultation		
8.3 Existence of champions		
9.1 Internal evaluation of the PPP		
9.3 External evaluation		

* 30. Do you identify some missing criteria presented in the sections 1 to 9 (available here) that can influence the quality OPERATIONALITY attribute, or do you have any other comments?

- No
- Yes (please specify which one(s))

Influence of the evaluation criteria on the ACCEPTABILITY attribute .
Acceptability: All relevant stakeholders demonstrate trust into the PPP, mutual understanding and willingness to collaborate. The objectives and outcomes of the PPP meet the stakeholder's expectations. Actors are satisfied with the repartition of resources. The PPPs have a societal legitimacy.

Please, indicate how the selected criteria influence the ACCEPTABILITY attribute and your degree of certainty about this influence

* 31. Does the following PPP process criteria influence the ACCEPTABILITY of the PPP?

	Level of influence	How confident are you with your answer?
1.4 Position of the partners regarding the common objective		
2.1 The specific interests of the different partners		
2.2 Repartition of benefits		
2.3 Repartition of the other outputs, ownership		
2.7 Repartition of the constraints		
3.1 Formalization of the PPP		
3.2 Knowledge of the terms of the PPP (contract) by the partners		
3.3 Endorsement by all partners		
3.4 Shared decision making process		
3.9 Funding & HR repartition		
4.2 Existence of organizational chart		
6.1 Confidence in other partner competencies		
6.2 Satisfaction of partners about their own competencies		
8.1 Willingness to collaborate		
8.2 Level of involvement of partners/mobilisation		
9.2 Choices of indicators for internal evaluations		
9.3 External evaluations		

* 32. Do you identify some missing criteria presented in the sections 1 to 9 (available here) that can influence the ACCEPTABILITY attribute, or do you have any other comments?

- ◯ No
- ◯ Yes (please specify which one(s))

Influence of the evaluation criteria on the ADAPTABILITY attribute.
Adaptability: PPP can adapt and evolve upon changes in governance modalities, organizational process and other structural modalities in order to best suit the changing environment.

Please, indicate how the selected criteria influence the ADAPTABILITY attribute and your degree of certainty about this influence.

* 33. Does the following PPP process criteria influence the ADAPTABILITY of the PPP?

	Level of influence	How confident are you with your answer?
3.7 Opportunities of private parties' involvement		
4.3 Potential other partners		
4.4 Inclusion of vulnerable groups		
4.6 Modalities of the PPP		
8.3 Existence of champions		
9.1 Internal evaluations		
9.2 Choices of indicators for internal evaluations		
9.3 External evaluations		

34. Do you identify some missing criteria presented in the sections 1 to 9 (available here) that can influence the ADAPTABILITY attribute, or do you have any other comments?

- No
- Yes (please specify which one(s))

Influence of the evaluation criteria on the INCLUSIVENESS attribute.
Inclusiveness: Relevant actors participate in governance mechanisms and decision making process. PPP provide a trustworthy environment where stakeholders can freely express their views and be heard, creating mutual understanding. New partners are considered if relevant. The vulnerable group(s) are taking into consideration.

Please, indicate how the selected criteria influence the INCLUSIVENESS attribute and your degree of certainty about this influence.

* 35. Does the following PPP process criteria influence the INCLUSIVENESS of the PPP?

	Level of influence	How confident are you with your answer?
2.2 Repartition of benefits		
2.3 Repartition of the other outputs, ownership		
2.7 Repartition of the constraints		
3.4 Shared decision making process		
3.7 Opportunities of private parties' involvement		
4.3 Potential other partners		
4.4 Inclusion of vulnerable group		
6.1 Trust in other partner competencies		

	Level of influence	How confident are you with your answer?
6.4 Accessibility and frequencies of trainings		
7.1 Consultation		
7.3 Communication with other parties and with end users		
7.4 Transparency		

* 36. Do you identify some missing criteria presented in the sections 1 to 9 (available here) that can influence the INCLUSIVENESS attribute, or do you have any other comments?

- No
- Yes (please specify which one(s))

PPP EVALUATION TOOL

End of the questionnaire

37. Do you have any comment to add?

Thank you for completing this questionnaire! As already mentioned, we will develop a second questionnaire dealing with the potential discrepencies between expert answers, after having analysed the results.

We will contact you within 2 weeks for the second part of this expert elicitation process to reach agreement between experts by adding clarification or modification in the criteria under validation.

You will receive feedback on this 1st part results in about 2 weeks.
