# Supplementary material for: An evaluation tool to strengthen the collaborative process of the public-private partnership in the veterinary domain
Source: PLoS One. 2021 May 28;16(5):e0252103. doi: 10.1371/journal.pone.0252103 (PMC8162688; doi:10.1371/journal.pone.0252103)
Supplement: S2 File — (PDF) [file pone.0252103.s002.pdf]

Presentation of the questionnaire

The purpose of this questionnaire is to elicit your expertise for a second round to fine a consensus on the selection of criteria and their influence on the quality attributes. This 2nd round of the expert elicitation will require you about 30min to respond.

Summary of the results of the 1st round

28 experts responded to the first questionnaire, and we thank you for your participation in this first round of the expert elicitation!

Methodology

To analyse the data, we used the methodology described below:
-The criteria from the 9 sections were validated if 85% or more of the participants considered it as relevant (if they were mentioned as « non-relevant » by less than 15% of the participants)
-The attributes were validated if 85% or more of the participants considered it as relevant.
-The level of influence was confirmed when more than 50 of the relative percentage of the participants selected the same level.

Key results of the part "validation of criteria"

-93% of the criteria (45 out 48) from the 9 sections were validated.
-Only 3 out of 48 criteria were considered as relevant by less than 85% of the participants (1 in section 3, 2 in section 4). Modifications/clarifications of those criteria are proposed for the 2nd round of the elicitation.
-Two new criteria, one in the section 4, one in the section 5, are proposed to be added to the evaluation tool and they are part of this 2nd round of expert elicitation.

Key results of the part "validation of attribute"

-The six attributes were validated

Key results of the part "influence of the criteria on the attributes"

-The level of influence of 14/16 of the criteria of the stability attribute, 6/8 of the criteria of the relevance attribute, 12/15 of the criteria of the operationality attribute, 15/17 of the criteria of the acceptability attribute, 7/8 of the criteria of the adaptability attribute and 11/12 of the criteria of the inclusiveness attribute were validated. The levels of influence of a criterion on an attribute which were not validated are part of the 2nd round of the expert elicitation.
-New criteria that influence some attributes were proposed by participants and are part of this 2nd round (11 in total)

This second questionnaire consists of 17 questions, divided in 3 parts.

Information on the expert (1 question)
Part 1 : modification and clarification of 3 criteria (3 questions)
Part 2 : proposition of two new criteria (2 questions)
Part 3: influence of the criteria on the attributes (11 questions)

You can download : (1) the questionnaire in pdf format, (2) the list and the definition of all the criteria, (3) the list and the definition of all the quality attributes here.

INFORMATION ON THE EXPERTS

* 1. Please enter your name and surname

Part 1. Modification and clarification of 8 criteria

In this part, we will ask you to validate or invalidate 8 criteria for which clarifications have been made

From the section 3 – governance and legal framework

* 2. The criterion **3.4 “shared decision making”** was considered as relevant by 82.14% of the participants. Thanks to the analysis of the comments of the participants, we propose here a new definition.

Past definition	Proposed new definition
Shared decision making with an equality in power relationship can represent a key success factor of the PPP	Shared decision making with an equality in power relationship can represent a key success factor of the PPP **to engage dialogue between partner. However, some decisions can be entirely the responsibility of one partner but that these should be made in consultation with the other PPP partners and with full transparency and understanding of how that decision impacts on all the relevant actors. When it comes to national public good, the public sector may have over riding powers.**

Is the new definition of the criterion 3.4 relevant when it comes to the assessment of the governance and legal framework of PPP (section 3) in the veterinary domain?

- ○ Relevant
- ○ Not relevant

If you selected the criterion as non-relevant, please specify

From the section 4 - planning and responsabilities

* 3. The criterion 4.3 “potential other partner” was considered as relevant by 82.14% of the participants. Thanks to the analysis of the comments of the participants, we propose here a new definition.

Past definition	New proposed definition
It could be useful/helpful to involve some other partners in the PPP for the stability of the initiative. They could have been identified by the partners	**A stakeholder mapping, to ensure that all the potential relevant or impacted (potential blocker) actors were identified and consulted, should have be made.** It could be useful/helpful to involve some other partners in the PPP for the stability of the initiative.

Is the criterion 4.3 relevant when it comes to the assessment of the planning and responsabilities of the PPP (section 4) in the veterinary domain?

- Relevant
- Not relevant

If you selected the criterion as non-relevant, please specify

* 4. The criterion 4.6 “modalities of PPP” was considered as relevant by 82.14% of the participants. Thanks to the analysis of the comments of the participants, we propose here a new definition.

Past definition	New proposed definition
4.6 Modalities of the PPP: By proposing a diversity of modalities of application to the partners, the PPP can satisfy a higher number of partners.	**4.6 Modalities of implementation of PPP activities: the modalities of implementation of PPP activities should be flexible to meet partners' needs.**

Is the criterion 4.6 relevant when it comes to the assessment of the planning and responsabilities of the PPP (section 4) in the veterinary domain?

- Relevant
- Not relevant

If you selected the criterion as non-relevant, please specify

From the section 5 - external factors and consequencies

* 5. The criterion 5.3 “organization of the private sector” was validated by the participants. Three participants mentioned that lack of organization of the public sector can also be a threat. We propose to extend the definition of the criteria 5.3.

Past definition	New proposed definition
5.3 Organisation of the private sector : Lack of organisation of the private sector could represent constraints for the proper implementation of the PPP. However, the PPP can also have the power to improve that organization, to improve the supply chain.	5.3 Organisation of the private a**nd/or the public sector** : Lack of organisation of the private sector (supply chain, market channel, producer association) **and/or the public sector (Veterinary Services)** could represent constraints for the proper implementation of the PPP. However, the PPP can also have the power to improve the organization of the public **and/or the private sector**

Is the criterion 5.3 relevant when it comes to the assessment of the external factors and consequencies of the PPP (section 5) in the veterinary domain?

- Relevant
- Not relevant

If you selected the criterion as non-relevant, please specify

From the section 6 - competencies and trainings

From the section 8 - collaboration

* 6. The criterion 8.3 “existence of champions ” was considered as relevant by 85% of the participants. Thanks to the analysis of the comments of the participants, we propose here a new definition.

Past definition	New proposed definition
8.3 Existence of champions: The existence of champion(s), who has experience in PPPs and promoting the well functionning of PPP at national, regional or local level, can represent a key success factor of the PPP.	**8.3 Willingness for capacity building in PPP : the existence of a senior or a capacity builder on PPPs best practices, at national, regional or local level, help to promote enable environment and good collaboration process**

Is the criterion 8.3 relevant when it comes to the assessment of collaboration process of the PPP (section 8) in the veterinary domain?

- Relevant
- Not relevant

If you selected the criterion as non-relevant, please specify

Part 2. Proposition of two new criteria

New criterion proposed on the section 4 planning and responsabilities

* 7. Is the new criterion 4.7 « joint work plan », proposed by one participant, relevant when it comes to the assessment of planning and responsabilities of the PPP (section 4) in the veterinary domain?

Criteria 4.7 “joint work plan”
A joint work plan about the activities to be implemented and on the roles and responsabilities of each partners should be co-elaborate by the partners. The elements of this work plan should be modify to allow an adaptability of the PPP.

- ◯ Relevant
- ◯ Non relevant

if you selected non relevant, please specify

New criterion proposed on the section 5 external factors and consequencies

* 8. Is the new criterion 5.5 “conflict of interest” proposed by one participant, relevant when it comes to the assessment of external factors and consequencies of the PPP (section 5) in the veterinary domain?

Criteria 5.5 “conflict of interest”
Potential conflict of interest are risks of PPP. The potential conflict of interests should be anticipated and all the procedures to avoid those potential conflict of interest should be planned.

- ◯ Relevant
- ◯ Non relevant

if you selected non relevant, please specify

Part 3. Influence of the criteria on the attributes

Influence of the evaluation criteria on the STABILITY attribute

STABILITY : The collaboration process in the PPP is stable in the time defined by the stakeholders, and thus despite the potential changing environment. The formalisation and endorsement of the agreement satisfied all relevant stakeholders.

The high level of inluence were validated for 12 criteria out of 16 (1.1, 1.5, 2.6, 3.1, 3.2, 3.3, 3.8, 4.5, 4.6, 5.3, 7.2, 7.4). The medium level of inluence were validated for 2 criteria out of 16 (4.3, 4.5).

For 2 criteria out of 16 (5.2, 5.4) the level of influence was not determined, as presented bellow *(please note that the criterion 5.2 'infrastructure' is not presented here as it will be merged with the critetion 5.1 'threat of the PPP')*:

	Relative percentage of participants who selected each level of influence
Level of influence	Criterion 5.4
High	39,39%
Moderate	39,40%
Weak	21,21%
No influence	0,00%

* 9. Based on the results of the other experts, can you select again the level of influence of the following criteria on the STABILITY of the PPP?

	Influence level	How confident are you with your answer?
5.4 Cost to the society (negative externalities)		

* 10. A new criterion was proposed by two participants, can you select its level of influence on the STABILITY of the PPP?

	Influence level	How confident are you with your answer?
9.1 Internal evaluation		

Influence of the evaluation criteria on the RELEVANCE attribute.

RELEVANCE: PPP strategy, modalities and activities are relevant regarding the main objective. The main objective is relevant regarding the context (epidemiological, institutional, environmental, societal). PPP represent a clear added-value to achieve the objective.

The high level of inluence were validated for 6 criteria out of 8 (1.3, 1.5, 2.1, 2.4, 2.5, 3.6) . The medium level of inluence were validated for 0 criteria.

For 2 criteria out of 8 (5.4, 6.5) the level of influence was not determined, as presented bellow (*please note that the criterion 6.5 'reinforcement of the VS through trainings' is not presented here as it was merged with the critetion 6.3 'organisation of trainings')*:

	Relative percentage of participants who selected each level of influence
Level of influence	Criterion 5.4
High	35,29%
Moderate	41,18%
Weak	20,59%
No influence	2,94%

* 11. Based on the results of the other experts, can you select again the level of influence of the following criterion on the RELEVANCE of the PPP?

	Level of influence	How confident are you with your answer?
5.4 Cost to the society (negative externalities)		

* 12. A new criterion was proposed by two participants, can you select its level of influence on the RELEVANCE of the PPP?

	Influence level	How confident are you with your answer?
2.2 repartition of the benefits		

Influence of the evaluation criteria on the OPERATIONALITY attribute.

Operationality: The governance of PPP is operational, and collaboration is effectively implemented to meet the main objective. Roles in PPP are adequately allocated to actors with regard to their mandates and competencies. Trainings are organised to be sure that stakeholders can fit their roles. The mechanisms for the resources allocation are defined. The resources are appropriate and available for the effective implementation of activities.

The high level of inluence were validated for 10 criteria out of 15 (1.1, 3.5, 3.8, 4.1, 5.2, 5.3 6.4, 7.1 ,9.1, 9.3) . The medium level of inluence were validated for 2 criteria (6.3, 2.8).

For 3 criteria out of 15 (4.5, 5.1, 8.3) the level of influence was not determined, as presented bellow:

	Relative percentage of participants who selected each level of influence		
Level of influence	Criterion 4.5	Criterion 5.1	Criterion 8.3
High	39,40%	48,65%	38,46%
Moderate	42,93%	45,95%	48,72%
Weak	12,63%	5,40%	12,82%
No influence	0,00%	0,00%	0,00%

* 13. Based on the results of the other experts, and based on the new definition of the criterion 8.3 can you select again the level of influence of the following criterion on the OPERATIONALITY of the PPP?

	Level of influence	How confident are you with your answer?
4.5 Predefined duration		
5.1 Threat of the PPP : anticipation and mitigation		
8.3 Willingness for capacity building in PPP		

Influence of the evaluation criteria on the ACCEPTABILITY attribute.

Acceptability: All relevant stakeholders demonstrate trust into the PPP, mutual understanding and willingness to collaborate. The objectives and outcomes of the PPP meet the stakeholder’s expectations. Actors are satisfied with the repartition of resources. The PPPs have a societal legitimacy

The high level of inluence were validated for 14 criteria out of 17 (1.4, 2.1, 2.2, 2.3, 2.7, 3.1, 3.2, 3.3, 3.4, 3.9, 6.1, 6.2, 8.1, 8.2). The medium level of inluence were validated for 1 criteria (4.2).

For 2 criteria out of 17 (9.2, 9.3) the level of influence was not determined, as presented bellow:

	Relative percentage of participants who selected each level of influence	
Level of influence	Criterion 9.2	Criterion 9.3
High	45,71%	34,29%
Moderate	45,71%	48,57%
Weak	8,57%	17,14%
No influence	0,00%	0,00%

* 14. Based on the results of the other experts, can you select again the level of influence of the following criterion on the ACCEPTABILITY of the PPP?

	Level of influence	How confident are you with your answer?
9.2 Choices of monitoring indicators for internal evaluation		
9.3 External evaluation		

* 15. Two new criteria were proposed by one participant, can you select their level of influence on the ACCEPTABILITY of the PPP?

	Influence level	How confident are you with your answer?
7.1 Consultation between partners		
7.3 Communication with other parties and with end users		

Influence of the evaluation criteria on the ADAPTABILITY attribute.

Adaptability: PPP can adapt and evolve upon changes in governance modalities, organizational process and other structural modalities in order to best suit the changing environment.

The high level of inluence were validated for 6 criteria out of 8 (3.7, 4.6, 8.3, 9.1, 9.2). The medium level of inluence were validated for 1 criteria (4.2).

For 1 criteria out of 17 (4.4) the level of influence was not determined, as presented bellow:

	Relative percentage of participants who selected each level of influence
Level of influence	Criterion 4.4
High	45,71%
Moderate	46,67%
Weak	11,43%
No influence	0,00%

* 16. Based on the results of the other experts, and based on the new definition of the criterion 8.3 can you select again the level of influence of the following criterion on the ADAPTABILITY of the PPP?

	Level of influence	How confident are you with your answer?
4.4 Inclusion of vulnerable group		

* 17. Six new criteria were proposed by participants, can you select their level of influence on the ADAPTABILITY of the PPP?

	Influence level	How confident are you with your answer?
2.6 Risk and constraints : anticipation and mitigation (proposed by 2 participants)		
2.8 Changes of practices (proposed by 2 participants)		
3.4 Shared decision making (proposed by 1 participant)		
6.3 Organisation of trainings (proposed by 2 participants)		
7.1 Consultation between partners (proposed by 1 participant)		
7.3 Communication with other parties and with end users (proposed by 1 participant)		

Influence of the evaluation criteria on the INCLUSIVENESS attribute.

Inclusiveness: Relevant actors participate in governance mechanisms and decision making process. PPP provide a trustworthy environment where stakeholders can freely express their views and be heard, creating mutual understanding. New partners are considered if relevant. The vulnerable group(s) are taking into consideration

The high level of inluence were validated for 11 criteria out of 12 (2.2, 2.3, 2.7, 3.4, 3.7, 4.4, 6.1, 6.4, 7.1, 7.3, 7.4). The medium level of inluence were validated for 1 criteria (4.3).

For 0 criteria out of 12 (6.4) the level of influence was not determined

* 18. A new criterion was proposed by two participants, can you select their level of influence on the INCLUSIVENESS of the PPP?

	Influence level	How confident are you with your answer?
8.4 actors acceptation of their own role		

End of the questionnaire

19. Do you have any comment to add?

Thank you for completing this questionnaire! Your inputs are really valuable to help us to developp the most apropriate evaluation tool of the PPP process.
You will receive feedback on the results of this two rounds!
