# Supplementary material for: An evaluation tool to strengthen the collaborative process of the public-private partnership in the veterinary domain
Source: PLoS One. 2021 May 28;16(5):e0252103. doi: 10.1371/journal.pone.0252103 (PMC8162688; doi:10.1371/journal.pone.0252103)
Supplement: S3 File — (DOCX) [file pone.0252103.s003.docx]

| Criteria | Quality attribute | |
| --- | --- | --- |
| Scoring the criteria | | Score & comment |

# Section 1: objective(s) of the PPP

| 1.1 Common objective(s) | **Operationality** | |
| --- | --- | --- |
| All partners (public and private actors) must co-construct and define the overall objective(s) to be achieved and the service to be delivered.   - Score 0: The partners do not agree on the definition of the common objective(s) to be achieved or the services to be provided. These objectives have not been co-constructed. - Score 1: The partners do not agree on the definition of the common objective(s) to be achieved (which have not been co-constructed) or on the services to be provided. - Score 2: The partners partly agree on the definition of the common objective(s) to be achieved but not on the services to be delivered - Score 3: All partners agree on the common objective(s) to be achieved which was co-constructed and on the services to be delivered. | |  |
| 1.2 Formalization of the common objective | **Stability** | |
| Level of formalization of the common objective of the PPP (MoU, Letter of Agreement, Oral consent etc.).   - Score 0: Absence of formalization or lack of formalisation, which hinders the proper process of PPPs. - Score 1: Formalized objectives warrant significant additional details. - Score 2: Formalized objectives warrant minor additional details. - Score 3: Well-detailed, fully formalized objectives of the PPP are written in a document recognized by all the partners. | |  |
| 1.3 Position of the partners regarding this common objective | **Acceptability** | |
| The common objective should be transparent and understood by each partner. It should satisfy each partner regarding his/her own strategies, needs and benefits.   - Score 0: Some of the partners are not satisfied with the common objective which lacks transparency. - Score 1: Only one type of partner is satisfied with the common objective. - Score 2: All types of partners are partly satisfied with the common objective and each partner understands the common objective. - Score 3: All types of partners are fully satisfied with the common objective which is transparent. | |  |
| 1.4 Added value of the PPP | **Stability; Relevance;** | |
| The PPP should represent an added value to reach the common objective(s) of the program (it has been considered that only one sector conduct this program, but in this case the objective could not be reach or would entail more difficulties).   - Score 0: This PPP does not provide a clear added value; on the contrary it represents a constraint to reach the common objective(s) of the program. - Score 1: The PPP is not a clear added value to reach the common objective. - Score 2: The PPP is an added value to reach the common objective even if this common objective can be reach by one partner alone. - Score 3: The PPP is a clear added value for the program and the common objective cannot be reached without the partner(s). | |  |

# Section 2. Specific interest / benefits

| 2.1. The specific interest of the different partners | **Relevance; Acceptability** | |
| --- | --- | --- |
| The different partners have specific interest and expected benefits in enrolling in the PPP. These specific interests should be explicit, transparent, formalized (if appropriate) and understood by the other partners. The specific interests shouldn’t hinder the achievement of the common objective.   - Score 0: Lack of identification of the specific interest of the different partners. - Score 1: The specific interests of some of the partners are not explicit and transparent. - Score 2: The specific interests of all the partners have been identified and discussed between partners. - Score 3: All partners’ specific interests are identified, formalized (if appropriate) and understood and accepted by the other partners. | |  |
| 2.2 Allocation of benefits and other outputs (ownership) | **Relevance; Acceptability; Inclusiveness** | |
| The PPP may have differing benefits for the public and private sectors. The partners should be satisfied with the allocation of benefits and other outputs (such as products, intellectual rights, property rights). The allocation of benefits and outputs should be formalized if appropriate. The profit and loss related to the program of each partner should be transparent.   - Score 0: One of the partners thinks that the other partner gets many more benefits than they do and is not satisfied with their own benefits. The allocation of PPP outputs has not been specified OR it does not satisfy some partner(s). - Score 1: The allocation of the specific benefits and PPP outputs has been partly discussed (not formalized) and does not satisfy some partners. - Score 2: The partners are partly satisfied with the allocation of benefits and of the other outputs. - Score 3: All partners are highly satisfied with the allocation of benefits and outputs, which is also properly formalized. | |  |
| 2.3. Achievement of goal(s) of the Veterinary Service | **Relevance** | |
| The PPP should help to reach the goal(s) previously defined by the Veterinary Services (VS).   - Score 0: The PPP does not help the VS to reach one of their goals; on the contrary it represents a constraint to reach one of their goals. - Score 1: The PPP does not help the VS to reach one of their goals. - Score 2: The goal(s) of the VS has been planned to be achieved through the help of the PPP. - Score 3: The goal(s) of the VS has been achieved through the help of the PPP. | |  |
| 2.4. Achievement of goal(s) of the private sector | **Relevance** | |
| The PPP can help to reach the goal(s) previously defined by the private sector.   - Score 0: The PPP does not help the private sector to reach one of their goals; on the contrary it represents a constraint to reach one of their goals - Score 1: The PPP does not help the private sector to reach one of their goals. - Score 2: The goal(s) of the private sector has been planned to be achieved through the help of the PPP - Score 3: The goal(s) of the private sector has been achieved through the help of the PPP | |  |

# Section 3. Risks and constraints

| 3.1. Risks and constraints of getting involved in the PPP | **Stability, Adaptability** | | |
| --- | --- | --- | --- |
| The different partners could have specific constraints/risks (financial, societal, etc.) in engaging in this PPP: these should be identified, discussed and understood by the partners.   - Score 0: Lack of identification of the constraints/risks of the different partners. - Score 1: A minority of partners have identified their potential constraints/risks. - Score 2: A majority of partners have identified their potential constraints/risks. - Score 3: All partners have identified their potential constraints/risks. | | |  |
| 3.2. Allocation of the constraints | **Acceptability; Inclusiveness** | | |
| The PPP may have differing constraints (financial, societal, etc.) for the public and/or private sectors, and the partners should be satisfied by the allocation of these constraints.   - Score 0: One partner thinks that the other partner has fewer constraints or risks than they do and is not satisfied with their own constraints or risks. - Score 1: One partner thinks that the other partner has far fewer constraints or risks than they do but is still satisfied with their own constraints or risks. - Score 2: The partners are partly satisfied with the distribution of constraints or risks. - Score 3: All partners are highly satisfied with the distribution of constraints or risks. | | |  |
| 3.3. Change of practices | **Operationality, Adaptability** | | |
| The achievement of the common objective may require a change in the practices (e.g. change or ban of the use of medicines, change in farming techniques, change in vaccination planning, etc.) of a specific population (veterinarians, technicians, farmers, etc.). These changes should be anticipated, accepted and accompanied if needed. The population concerned by the changes should be consulted from the beginning of the process.   - Score 0: The potential changes of practices of population(s) have not been anticipated. - Score 1: The potential changes of practices of population(s) have been anticipated but not discussed with the concern population. - Score 2: The potential changes of practices of population(s) have been anticipated, discussed with the concerned population but not accompanied. - Score 3: The potential changes of practices of population(s) have been anticipated, discussed and accepted from the beginning of the process by the concerned population and accompanied if needed. | | |  |
| Negative costs to the society | **Stability; Relevance** | | |
| Every initiative can carry some negative societal cost (e.g. constraints on a category of partners), economic cost (e.g. financial competitiveness with other partners, competitiveness for resource) or environmental cost (contamination)/ biodiversity cost (loss of wild or domestic animal or plant biodiversity). These costs should be anticipated in order to be minimized.   - Score 0: The partners pay no attention to negative costs to the society of the PPP. - Score 1: The partners have partly identified negative costs to the society. - Score 2: The partners have partly identified negative costs to the society and take them into account in the PPP modalities. - Score 3: The partners have identified all the negative costs to the society and found a way to overcome them. | | |  |
| 3.5 Conflicts of interest | | **Stability, Acceptability** | |
| Potential conflicts of interests pose potential risks for a PPP. The potential conflicts of interests should be anticipated and all the procedures should be planned to avoid these potential conflicts of interests.   - Score 0: Conflicts of interests threaten the PPP. - Score 1: Potential conflicts of interests have not been identified. - Score 2: Potential conflicts of interests have been identified but a clear procedure to avoid them has not been put in place. - Score 3: Potential conflicts of interests have been identified and all the procedures to avoid them are put in place. | | |  |

# Section 4. Analysis of the context and external factors

| 4.1 Relevance of common objective and of strategy regarding the context | **Relevance** | | | |
| --- | --- | --- | --- | --- |
| The common objective(s) should be relevant regarding the health (public and animal health, food safety), socio-economic, environmental and institutional (breeding policy, local politics, national politics etc.) context.   - Score 0: The epidemiological, socio-economic, environmental and institutional contexts have not been analysed. - Score 1: The contexts have been analysed but some major contradictions have been identified between the common objective and the contexts. - Score 2: Some minor contradictions have been identified between the common objective and the contexts. - Score 3: The common objective of the PPP is fully coherent with the all the dimensions of the analysed contexts and serves the common good. | | |  | |
| 4.2. International, regional, national and local laws | | **Operationality** | | |
| Legal obligations, laws and constraints from international organizations, regions, the country or localities are understood and properly applied by all partners and a public partner is responsible to ensure application of the laws. The public partner should ensure that the PPP is lawful and that any legal obligations or constraints are understood and properly implemented by all parties.  Potential need for regulatory and / or policy changes that might be required to implement the PPP should be considered and anticipated.   - Score 0: International, regional, national and local guidance have not been identified and some guidelines are not respected. - Score 1: Some major discrepancies are identified between the objective and purpose of the PPP or the actions of some partners and international, regional, national and local guidance. - Score 2: Some minor discrepancies are identified between the objective and purpose of the PPP or the actions of some partners and regional, national and local guidance. - Score 3: The objective and purpose of the PPP and the actions of the partners are coherent with the international, regional, national and local guidance. | | |  | |
| 4.3 Potential threats of the PPP and mitigation | | **Stability; Operationality** | | |
| Some external factors related to the context (epidemiological such as a pandemic, institutional such as political change or insecurity, socio-economic such as fluctuating market or civil society expectations, environmental such as extreme weather risks etc.) can threaten the stability of the PPP. For example, lack of appropriate infrastructures (such as road, water, electricity, etc.) could represent constraints for the proper implementation of the PPP. However, the PPP can also have the power to remediate these failures, to respond to these constraints. These should be anticipated and mitigation strategy for these potential threats put in place.   - Score 0: The potential threats have not been identified. - Score 1: The potential threats have been partially identified but the strategy to overcome them has not been discussed. - Score 2: The potential threats have been analysed and a strategy to overcome them has been discussed but not implemented. - Score 3: The potential threats have been analysed and the strategy of implementation of PPP activities is based on the prevention of these potential risks. | | |  | |
| 4.4 Organisation of private and public sectors | | **Stability; Operationality** | | |
| Lack of organization of the private sector (supply chain, market channel, producer association) and/or the public sector (official Veterinary Services) could represent constraints for the proper implementation of the PPP. However, the PPP can also aim to improve the organization of the public and/or private sector.   - Score 0: Private and/or public sector organization is a major constraint and the PPP cannot improve this organization. - Score 1: Private and/or public sector organization is a minor constraint and the PPP cannot improve this organization. - Score 2: Private and/or public sector organization is not a constraint for the PPP OR private and/or public sector organization is a minor constraint and the PPP can improve this organization. - Score 3: The PPP is a strength to improve private and/or public sector organization. | | | |  |
| 4.5 Analysis of pre-existing PPPs | | **Relevance** | | |
| If other PPP in the same geographical area or with similar objectives exist, the analysis of their key success factors, obstacles and outcomes could be helpful for implementing the good process practices of this PPP.   - Score 0: Other PPP have not been identified. - Score 1: Other relevant PPP have been identified but not analysed. - Score 2: Other relevant PPP have been identified and analysed. - Score 3: Other relevant PPP have been identified, analysed, and the partners from the different PPP shared their experiences about key success factors, obstacles and outcomes. | | |  | |

# Section 5. Governance of the PPP

| 5.1. Formalization of the PPP (contracts, sanitary mandate) | **Stability; Acceptability** | |
| --- | --- | --- |
| The terms of the rationale of the PPP should be formalized if appropriate, either in a formal contract or in an alternative form appropriate to the PPP (MoU, Letter of Agreement, Oral consent etc.). It should be considered that a high level of formalization is not necessarily the most appropriate (for example the high degree of formality of an early collaborative PPP would put off potential partners).   - Score 0: There is no contract/text or agreement and this hinders the proper process of PPP. - Score 1: There is an unofficial agreement which would warrant a greater level of formalization to favour a better process. - Score 2: There is an official agreement but it is not signed by all partners (?) - Score 3: The level of formalization of the PPP is fully adapted and allows for a proper process. | |  |
| 5.2. Knowledge of the terms of the PPP (contract) and endorsement by all the partners | **Stability; Acceptability** | |
| The different partners should be aware of the terms of the contract and understand them all. The documents where the terms of the PPP are formalized (if appropriate) are endorsed by all partners from different sectors.   - Score 0: There is no contract/text OR there is a contract/text but some partners are not aware of it, and it is endorsed by none of the partners or only from one type of partner. - Score 1: The terms of the agreement are understood and endorsed only by some of the partners (less than half). - Score 2: The terms of the agreement are partially understood by the partners and are endorsed by most of the partners. - Score 3: The terms of the agreement are fully understood and endorsed by all relevant partners. | |  |
| 5.3. Shared decision-making process | **Acceptability; Adaptability; Inclusiveness** | |
| Shared decision making with equality in the power relationship can represent a key success factor of the PPP, recognizing that some decisions can be entirely the responsibility of one partner. However, such decisions should be made in consultation with the other PPP partners and with full transparency and understanding of how that decision impacts all the relevant actors. It should be considered that shared decision making is time-consuming and costly, and may not be necessary for all decisions.   - Score 0: All the decisions are only taken by one type of partner and imposed on the other partners. - Score 1: Few decisions are taken in collaboration and there is a need to set up a mechanism for shared decision making. - Score 2: A mechanism for shared decision making is set up but could be improved. - Score 3: A mechanism for shared decision making is set up and the partners are satisfied with it. | |  |
| 5.4. Opportunities of private parties’ involvement | **Adaptability; Inclusiveness** | |
| If the proposal is initiated by the public party, it should ensure that relevant private partners have equal opportunities for engagement in a new PPP, respecting the country market rules. The public sector should propose a transparent call for tender process. If a proposal is initiated by the private sector, fair access is still a consideration for the public sector, subject to the specificity of the project and the laws of the country. As a minimum, the public sector should ensure that all relevant private sector actors are aware of the possibility of engaging in a PPP.   - Score 0: There was no call for tender, a direct contract was formed with one private partner previously selected AND/OR the PPP was initiated by the private sector and the public sector did not communicate to other private sector actors. - Score 1: There was an oriented call for tender and only a few of the relevant private sector actors were aware of the possibility of engaging in a PPP AND/OR the PPP was initiated by the private sector and the public sector communicated in a non-transparent manner to other private sector actors. - Score 2: There was an oriented call for tender but most of the relevant private sector actors were aware of the possibility of engaging in a PPP AND/OR the PPP was initiated by the private sector and the public sector weakly communicated the possibility of engaging in a PPP to other private sector actors. - Score 3: Open transparent call for tender and all relevant private sector actors are aware of the possibility of engaging in a PPP AND/OR the PPP was initiated by the private sector and the public sector communicated the possibility of engaging in a PPP to other private sector actors in a transparent manner. | |  |
| 5.5. Funding & human resource availability | **Stability; Operationality** | |
| Funding and human resources (HR) should be available and sufficient. If an external source is providing money, the PPP should plan how to be autonomous and viable when the other source stops. The stability of human resources must be anticipated, as some people may evolve in their career and no longer be able to fulfil their role in the PPP.   - Score 0: The question of funding and HR availability is a major constraint for the different partners and hinders the proper conducting of PPP activities. - Score 1: The question of funding and HR availability is a regular constraint in conducting the PPP’s activities, or they depend entirely on an external source (catalysers e.g. UN, Private foundations etc…) with no plan to become autonomous. - Score 2: The conducting of the PPP is only weakly constrained by funding and HR availability or it depends partly on an external source with a plan to become autonomous in the short term. - Score 3: The different partners are fully satisfied with the funding and HR availability for conducting their activities in the PPP, the PPP is financially viable. | |  |
| 5.6. Funding and human resource allocation | **Acceptability** | |
| The allocation of funding and HR should be planned in advance and agreed on by the partners.   - Score 0: The question of funding and HR allocation is a major concern for some partners and hinders the proper conducting of its activities. - Score 1: The question of funding and HR allocation is a regular concern for some partners. - Score 2: Some partners are not totally satisfied by the funding and HR allocation between the different partners. - Score 3: All partners are fully satisfied with the funding & HR allocation between the different partners. | |  |
| 5.7. Compatibility with the Veterinary Services mandate | **Relevance** | |
| The public partner(s) must ensure that the service(s) to be delivered falls within their VS statutory or political mandate and fulfils the intention of that mandate. The mandate should not be weakened and the public sector must continue to bear full responsibility for the VS mandate with complete independence.   - Score 0: The services to be delivered go against the intention of the VS mandate or the role of the private encroaches on the role of the public. - Score 1: The services to be delivered do not take into account the intention of the Veterinary Services mandate. - Score 2: The services to be delivered are partly aligned and partly help to fulfil the intention of the Veterinary Services mandate. - Score 3: The services to be delivered are totally aligned and help to fulfil the intention of the Veterinary Services mandate. | |  |

# Section 6. Planning and responsibilities of the PPP

| 6.1. Division of roles and responsibilities | | **Operationality; Acceptability** | | |
| --- | --- | --- | --- | --- |
| The role of each partner should be properly defined. Formalisation of the partner’s areas of action in the PPP should be specified in the contract if appropriate, i.e. the tasks they are assigned regarding collaboration and coordination of PPP. An organizational chart of the PPP can provide a useful element to understand who depends on whom, who decides for whom.   - Score 0: The role and responsibility of the PPP partners are not properly defined and this hinders the proper process of the PPP. - Score 1: The role and responsibility of the PPP partners are partly defined but lack major details. - Score 2: The role and responsibility of the PPP partners are set out in a document but the definitions sometimes lack clarity, details or the description of areas of responsibility of some partners. - Score 3: The role and responsibility of the PPP partners are framed by a document (official document if appropriate) leaving no ambiguity in the relations between them. | | | |  |
| 6.2. Potential other partners | **Stability; Adaptability; Inclusiveness** | | | |
| Stakeholder mapping, to ensure that the relevant or impacted (potential blocker) actors have been identified and consulted, should be carried out regularly during the PPP. Some of the identified actors could be involved in the PPP to ensure the stability of the initiative and to favour positive results.   - Score 0: No stakeholder mapping has been carried out. Some relevant partners are missing but have not been properly identified. - Score 1: No stakeholder mapping has been carried out. Some partners have been identified as missing but no plan is designed to integrated them in the partnership. - Score 2 : Incomplete stakeholder mapping has been carried out. Some partners have been identified as missing but no plan is designed to integrated them in the partnership. - Score 3: Complete stakeholder mapping has been carried out, and is regularly updated. If appropriate, the relevant partners have already been identified and planned to be included in the PPP. Or, the questions has been raised but the partners agree that no other partners are needed. | | | |  |
| 6.3. Inclusion of vulnerable group | | | **Inclusiveness; Adaptability** | |
| PPPs should enhance equity in terms of their outcomes (economy, health, well-being etc.). This can be done by truly involving all the beneficiaries, including the vulnerable group (indigenous, women, young people, etc.) during the conception phase of the PPP to consider their interest, or at a minimum by inviting them to meetings or workshops.   - Score 0: The PPP favours the exclusion of vulnerable groups. - Score 1: The PPP does not consider the interest of vulnerable groups. - Score 2: The PPP considers the interest of vulnerable groups and invites some of their representatives to meetings or workshops. - Score 3: The PPP aim to enhance equity in terms of their outcomes (economy, health, well-being etc.) and truly involve all the beneficiaries, including vulnerable groups (indigenous, women, young people, etc.) during the conception phase of the PPP to consider their interest. | | | |  |
| 6.4. Defined duration | | | **Operationality; Stability*;*** | |
| The duration of the partnership should be predefined by both types of partner, with the possibility of extending the period or renewing the PPP if appropriate under predefined renewal conditions (e.g. if deemed appropriate following joint evaluation).   - Score 0: The duration term of the PPP has not been discussed and defined. - Score 1: The duration term is partly defined OR the duration term is fixed, without the possibility of extending the PPP. - Score 2: The duration of the PPP is predefined and agreed by both partners, but the conditions to extend the period have not been defined or are unclear. - Score 3: The duration of the PPP is predefined and agreed by both partners, with the possibility of extending the period under predefined renewal conditions. | | | |  |
| 6.5. Modes of implementation of PPP activities | | | **Stability; Adaptability** | |
| The implementation modes for PPP activities should be flexible to meet partners' needs.  By proposing a diversity of modes of implementation for activities, the PPP can satisfy a higher number of partners.   - Score 0: A single mode of implementation is proposed to the partners. - Score 1: A dominant mode of implementation is proposed to the partners. - Score 2: Several modes of implementation are proposed to the partners but still do not satisfy the partners’ need. - Score 3:Several modes of implementation are proposed to the partners and satisfy the partners’ need. | | | |  |
| 6.6. Joint work plan | | | **Operationality; Adaptability** | |
| A detailed joint work plan for the activities to be implemented and the roles and responsibilities of each partners regarding those activities should be jointly drawn up by the partners. The elements of this work plan should be modifiable to enable PPP adaptability.   - Score 0: No joint work plan - Score 1: There is a work plan but it has been devised by one type of partner and does not satisfy all the partners. - Score 2: A work plan has been devised but could be improved. - Score 3: A detailed joint work plan has been devised, with elements being modifiable to enable PPP adaptability. | | | |  |

# Section 7. Competencies and trainings

| 7.1 Confidence in other partners’ competencies and satisfaction of partners about their own competencies | **Acceptability; Inclusiveness** | | |
| --- | --- | --- | --- |
| The partners should feel confident about their partner competencies to fulfil the common objective(s). The different partners should be satisfied with their own competencies to reach the common objective(s); the partners must be able to inscribe their role.   - Score 0: Partners don’t trust their partner competencies to reach the common objective; and the partners are not satisfied with their competencies, nor do they feel confident about their abilities to inscribe the roles. - Score 1: Partners don’t trust their partner competencies to reach the common objective; or the partners are not satisfied with their competencies and don’t feel confident about their abilities to inscribe the roles. - Score 2: The partners do not fully trust their partner competencies but are confident that those competencies can improve (through training for example). The partners are partly satisfied with their competencies and are confident that these competencies can improve. - Score 3: All the partners trust their partner competencies to reach the common objective(s). The partners fully trust their own competencies to reach the overall objective. | | |  |
| 7.2 Organisation of trainings and capacity building | | **Operationality, Relevance, Adaptability** | |
| Well designed and well planned trainings should be organized for operating partners if needed. An initial capacity assessment can be made to plan the trainings. Funding for trainings should be planned. The Veterinary Service, as well as private technical skills can be reinforced by the PPP through organized training.   - Score 0: No training for operating partners involved in collaborative activities is planned. The VS do not participate in any training and this hinders the proper process of the PPP. - Score 1: Trainings for operating partners involved in collaborative activities are planned but more trainings are required. - Score 2: Trainings for operating partners involved in collaborative activities are planned/have been conducted but the partners are not fully satisfied with the content. - Score 3: Training for operating partners involved in collaborative activities is fully designed and planned in detail and the concerned partners are fully satisfied with the content. The VS benefit from trainings (if appropriate) which build their capacity and reinforce the trust of its partner. | | |  |
| 7.3 Accessibility and frequency of trainings | | **Operationality; Inclusiveness** | |
| The training organized should be at an appropriate frequency and should be accessible to all operating partners, to all partners that feel the need to improve their competencies.   - Score 0: Trainings organized are not accessible for the majority of the operating partners. - Score 1: Trainings organized are accessible to everyone but only a few of the partners participate and the frequency is not appropriate. - Score 2: Trainings are organized in a relevant timeframe and most of the partners participate . - Score 3: All relevant partners participate regularly in the trainings. | | |  |

# Section 8. Communication and transparency of the PPP

| 8.1. Internal communication | **Operationality; Acceptability; Adaptability; Inclusiveness;** | |
| --- | --- | --- |
| The PPP must have an agreed internal communication strategy. The frequency of meetings is to be assessed according to the need of the partners.   - Score 0: The partners have no mechanisms for internal communication with each other. - Score 1: The partners maintain informal channels for internal communication with each other; meetings are rarely and insufficiently organized for the purposes of the partners. - Score 2: The partners maintain a formal internal communication mechanism with each other, meetings are organized but at a frequency that appears insufficient to meet the needs of the partners. - Score 3: Meetings are regularly organized, the interested parties maintain a formal internal communication mechanism with each other and actively consult with and solicit feedback regarding proposed and current activities. | |  |
| 8.2. Agreement in resolution modalities in case of conflict | **Stability** | |
| A manner to resolve potential conflict(s) between partners should be identified: which partner/jurisdiction to contact, how to resolve this conflict?   - Score 0: No potential conflict resolution strategy. - Score 1: No official potential conflict resolution strategy has been developed but an informal strategy has proved sufficient for the moment. - Score 2: A potential conflict resolution strategy has been developed but it is not known by all the partners. - Score 3: An official potential conflict resolution strategy has been developed. Every partner knows whom to address, and what to do in case of conflict. | |  |
| 8.3 Communication with other parties, political entities and end users | **Acceptability, Adaptability, Inclusiveness** | |
| The partners should keep other parties informed (such as beneficiaries and end users, actors impacted) in a transparent, effective and timely manner, of PPP activities and results, since the beginning of the process. Furthermore, the partners should inform the executive and political level about PPP activities and results in a transparent, effective and timely manner, in order to be able to discuss the potential need for a change of regulations and to promote the positive results of the PPP.   - Score 0: The PPP have no mechanism in place to inform other parties of PPP activities and results. - Score 1: The PPP have informal communication mechanisms with other parties. - Score 2: The PPP maintain an official contact point for communication but it is not always up-to-date in providing information. - Score 3: The PPP contact point for communication provides up-to-date information, accessible via the Internet and other appropriate channels, on activities and results. | |  |
| 8.4 Transparency | **Inclusiveness; stability** | |
| All parties must ensure that the actions of the PPP are developed with appropriate transparency for all stakeholders at every level (allocation of outputs, of benefits, allocation of risk, modalities of action, activities of each partner etc.).   - Score 0: The transparency is insufficient at most levels. - Score 1: The transparency is sufficient at some levels. - Score 2: The transparency is sufficient in most levels. - Score 3: The transparency of the actions developed and the process of collaboration in the PPP is appropriate at all levels. | |  |

# Section 9. Collaboration in the PPP and satisfaction of the partners?

| 9.1. Willingness to collaborate and partners’ acceptance of their own roles | **Acceptability; Inclusiveness** | |
| --- | --- | --- |
| The partners should be happy/satisfied to collaborate with their partners and the PPP must have an agreed stakeholder engagement, which includes an appropriate approval process (formalisation of rationale behind the willingness to collaborate in this PPP).  The different partners should be satisfied with their own roles in the partnership and their tasks and with the recognition of their role by the other partners.   - Score 0: The partners are unsatisfied with collaborating with the other partner(s) and their willingness to collaborate has never been formalized; the partners are really unsatisfied with their own role (either because they seek more responsibilities, because their role is not socially recognized etc.) - Score 1: Only some partners are fully satisfied with collaborating with the other partner(s). Some of the partners are satisfied with their own role and with the recognition of their roles by the other partner. - Score 2: Most of the partners are fully satisfied with collaborating with the the other partner(s). Most of the partners are satisfied with their own role and with the recognition of their roles by the other partner. - Score 3: All the partners are fully satisfied with collaborating with their partners and their willingness to collaborate is formalized. All the partners are satisfied with their own role, and with the recognition of their roles by the other partner. | |  |
| 9.2. Level of involvement of partners | **Acceptability** | |
| Partners should be satisfied about the engagement of other partners in their assigned areas of action, role and responsibilities in the PPP.   - Score 0: None of the partners are satisfied with the involvement of the other partner(s). - Score 1: Some partners are satisfied or partly satisfied with the involvement of the other partner(s). - Score 2: Most partners are satisfied or partly satisfied with the involvement of the other partner(s). - Score 3: All the partners are fully satisfied with the level of involvement of the other partner(s). | |  |
| 9.3. Capacity building in PPPs and/or existence of champion(s) | **Operationality; Adaptability** | |
| The existence of senior capacity builder(s) for PPP best practices, and/or champion(s) (individuals with strong communication skills who are knowledgeable and enthusiastic about the PPP), at regional, national, or local level, may help to promote an enabling environment and a good collaboration process.   - Score 0: There are no champions and no seniors. - Score 1: There are no champions and no seniors but a process of recruitment and training has been initiated. - Score 2: There is a champion and/or a senior who partly promotes an enabling environment and a good collaboration process. - Score 3: Both senior(s) and champion(s) promote an enabling environment and a good collaboration process. | |  |

# Section 10. Monitoring and evaluation of the PPP

| 10.1. Internal monitoring of the PPP | **Operationality; Stability; Adaptability** | |
| --- | --- | --- |
| The different PPP partners should frequently monitor the progress of the program and discuss the main conclusion and ways to improve the PPP. They should be able to adapt the process and activities regarding the results of internal monitoring.   - Score 0: No internal monitoring has been done, nor is planned. - Score 1: Internal monitoring has been done by only one type of partner and the other partners are not kept informed, internal monitoring does not cover all the relevant areas of the PPP. - Score 2: Internal monitoring has been done by only one type of partner and the other partners are kept informed, the internal monitoring covers almost all the relevant areas of the PPP. - Score 3: Internal monitoring is done regularly by all the relevant partners, and the results allow for positive change. | |  |
| 10.2. Agreed indicators for joint internal monitoring | **Acceptability; Adaptability** | |
| The partners must agree on how the PPP is monitored, and on the choices of indicators for internal evaluation. The indicators should be SMART (specific, achievable, measurable, relevant, time-bound).These results indicators can be linked to the strategies of the country, of the Veterinary Services or of other private sectors in order to strengthen the visibility of PPP activities (for example livestock development strategies, sustainable development goals, employment).   - Score 0: Most partners are not satisfied with the indicators developed or no indicators have been developed. - Score 1: Indicators have been developed but only one sector is satisfied with the indicators. - Score 2: Indicators have been developed and validated by the PPP partners, but not all the partners agree with the methodology of internal evaluation and its frequency. - Score 3: The indicators have been developed in conjunction with all the partners. The indicators developed are SMART and address all areas of PPP ). All the partners agree with the methodology of internal evaluation and its frequency. | |  |
| 10.3. External evaluations | **Operationality; Acceptability; Adaptability** | |
| External evaluation helps to promote positive changes in the PPP. Partners must agree on how the PPP is evaluated, and on the choices of indicators for external evaluation.   - Score 0: No external evaluation of the PPP has been performed. - Score 1: The PPP has been evaluated but it goes back quite far in time AND / OR the method used is very incomplete or unrecognized and did not help to favour positive change. - Score 2: The PPP has already been the subject of several evaluations but their frequency needs to be improved and / or the methodology used is incomplete. - Score 3: The PPP is subject to external evaluations according to a recognized and complete methodology which helped to favour positive changes, and the partners are satisfied by the frequency. | |  |
